# KLHL17/Actinfilin, a brain-specific gene associated with infantile spasms and autism, regulates dendritic spine enlargement

**DOI:** 10.1186/s12929-020-00696-1

**Published:** 2020-12-01

**Authors:** Hsiao-Tang Hu, Tzyy-Nan Huang, Yi-Ping Hsueh

**Affiliations:** Institute of Molecular Biology, Academia Sinica, 128, Academia Road, Section 2, Taipei, 11529 Taiwan, Republic of China

**Keywords:** Actinfilin, F-actin cytoskeleton, Infantile spasms, Kelch-like 17, Dendritic spine enlargement

## Abstract

**Background:**

Dendritic spines, the actin-rich protrusions emerging from dendrites, are the subcellular locations of excitatory synapses in the mammalian brain. Many actin-regulating molecules modulate dendritic spine morphology. Since dendritic spines are neuron-specific structures, it is reasonable to speculate that neuron-specific or -predominant factors are involved in dendritic spine formation. KLHL17 (Kelch-like 17, also known as Actinfilin), an actin-binding protein, is predominantly expressed in brain. Human genetic study has indicated an association of KLHL17/Actinfilin with infantile spasms, a rare form of childhood epilepsy also resulting in autism and mental retardation, indicating that KLHL17/Actinfilin plays a role in neuronal function. However, it remains elusive if and how KLHL17/Actinfilin regulates neuronal development and brain function.

**Methods:**

Fluorescent immunostaining and electrophysiological recording were performed to evaluate dendritic spine formation and activity in cultured hippocampal neurons. Knockdown and knockout of KLHL17/Actinfilin and expression of truncated fragments of KLHL17/Actinfilin were conducted to investigate the function of KLHL17/Actinfilin in neurons. Mouse behavioral assays were used to evaluate the role of KLHL17/Actinfilin in brain function.

**Results:**

We found that KLHL17/Actinfilin tends to form circular puncta in dendritic spines and are surrounded by or adjacent to F-actin. *Klhl17* deficiency impairs F-actin enrichment at dendritic spines. Knockdown and knockout of KLHL17/Actinfilin specifically impair dendritic spine enlargement, but not the density or length of dendritic spines. Both N-terminal Broad-Complex, Tramtrack and Bric-a-brac (BTB) domain and C-terminal Kelch domains of KLHL17/Actinfilin are required for F-actin remodeling and enrichment at dendritic spines, as well as dendritic spine enlargement. A reduction of postsynaptic and presynsptic markers at dendritic spines and altered mEPSC profiles due to *Klhl17* deficiency evidence impaired synaptic activity in *Klhl17*-deficient neurons. Our behavioral assays further indicate that *Klhl17* deficiency results in hyperactivity and reduced social interaction, strengthening evidence for the physiological role of KLHL17/Actinfilin.

**Conclusion:**

Our findings provide evidence that KLHL17/Actinfilin modulates F-actin remodeling and contributes to regulation of neuronal morphogenesis, maturation and activity, which is likely relevant to behavioral impairment in *Klhl17*-deficient mice.

*Trial registration* Non-applicable.

## Introduction

Dendritic spines, tiny protrusions emerging from dendrites, are specific subcellular structures for excitatory synapses in mammalian brains [1]. Dendritic spines are mainly supported by F-actin [2, 3]. Thus, many F-actin binding proteins and/or regulators are involved in controlling dendritic spine morphology and density [4, 5]. Since dendritic spines are neuron-specific, it is reasonable to assume that neuron-specific F-actin regulators are involved in dendritic spinogenesis. Kelch-like 17 (KLHL17, also known as *Actinfilin*/*AF*; hereafter, *AF* and *KLHL17* are interchangeable in this report) is a neuron-specific F-actin binding protein [6, 7]. An analysis of biochemical subcellular fractions indicated the presence of KLHL17/AF in the postsynaptic density fraction, albeit at a low level [6], and KLHL17/Af has been shown to regulate F-actin remodeling [8]. However, it remains unclear if KLHL17/Af controls dendritic spine morphology or plasticity.

**Fig. 1:**
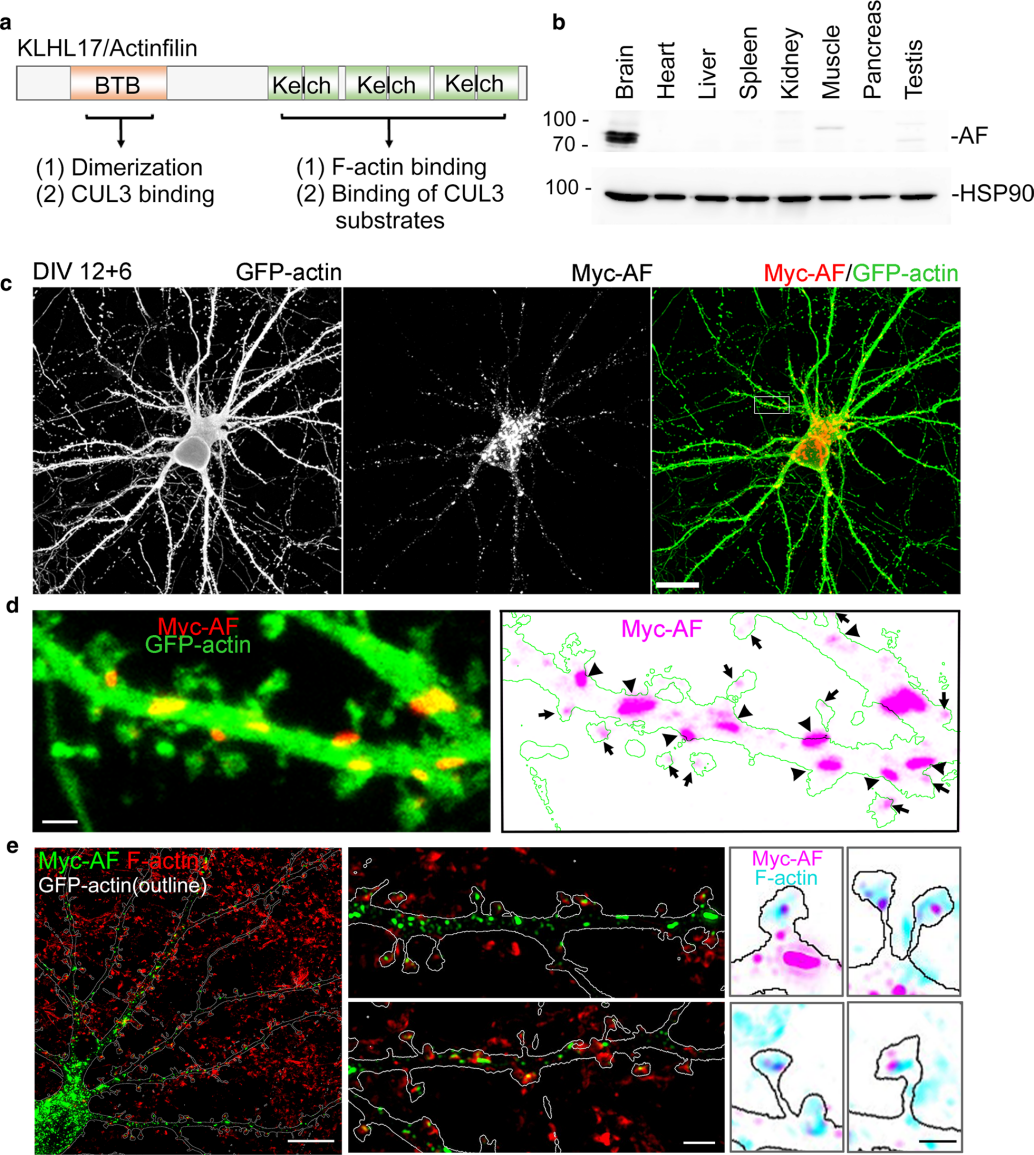
KLHL17/AF, a brain-specific protein, is colocalized with or adjacent to F-actin in mature neurons.** a** Functional domains of KLHL17/AF. Both the N- and C-terminal regions of KLHL17/AF have two functions, as indicated. **b** Expression of KLHL17/AF in diverse adult mouse tissues (3-months-old). A total of 20 μg of tissue lysates from each sample was analyzed by immunoblotting using AF and HSP90 antibodies. **c–e** Subcellular distribution of KLHL17/AF in mature neurons. Cultured hippocampal neurons were co-transfected with a Myc-tagged AF and GFP-actin construct at 12 DIV and immunostained at 18 DIV. **c**, **d** Confocal images. **c** Immunoreactivity of KLHL17/AF, as revealed by Myc antibody, shows punctate signal in the soma and dendrites of mature cultured neurons. **d** Locally enlarged dendrite (white box in **c**) to highlight the distribution of KLHL17/AF in dendrites and dendritic spines. Dendrite morphology was outlined by the signals of GFP-actin. Arrows indicate AF localized in the dendritic spine. Arrowheads indicate AF localized at the base of dendritic spines. **e** Super-resolution images of KLHL17/AF and F-actin immunoreactivities in a mature cultured neuron. AF is colocalized with or adjacent to F-actin at dendritic spines. F-actin signal was revealed by phalloidin-Alexa Fluor-405. Scale bars: **c** 20 μm; **d** 2 μm; **e** whole cell, 10 μm; enlarged dendrite segment, 2 μm; enlarged dendritic spine, 1 μm

Similar to other Kelch-like proteins, KLHL17/AF contains a Broad-Complex, Tramtrack and Bric-a-brac (BTB) domain at its N-terminal region and six Kelch domains at its C-terminal region (Fig. 1a). The N-terminal region of KLHL17/AF is involved in homo-oligomerization and interaction with the Cullin 3 (CUL3) ubiquitination complex [9]. The C-terminal Kelch domains interact with F-actin and substrates of CUL3-dependent E3 ligase [6, 9]. Thus, KLHL17/AF may perform two functions: one is to interact with actin cytoskeleton and control F-actin remodeling [8]; the other is to regulate ubiquitin-dependent protein degradation [9].

Human genetic analysis has suggested a link between KLHL17/AF and infantile spasms (IS), also known as West syndrome. IS is a type of early onset seizure [10–13] associated with autism spectrum disorders and mental retardation [14, 15]. In addition to *KLHL17/AF*, several genes have been linked to IS including *GRIN1*, *GRIN2A*, *MEF2C*, *STXBP2* and *MAGI2* [16]. These candidate genes are highly relevant to synaptic responses, so synaptic dysregulation or dysfunction is likely critical to IS. It further implies a role for KLHL17/AF in synaptic function.

In this report, we used cultured hippocampal neurons to explore if KLHL17/AF regulates dendritic spine formation and activity. We combined both knockdown and knockout approaches to manipulate KLHL17/AF protein levels and found that KLHL17/AF is essential for establishing the size of dendritic spine heads and synaptic activity. Behavioral analysis was also performed to characterize *Klhl17*-deficient mice. Moreover, truncated constructs consisting solely of the N- or C-terminal half of KLHL17/AF protein were further used to confirm that both domains are required for its function in controlling F-actin dynamics and dendritic spine morphology. Our study provides compelling evidence that KLHL17/AF controls dendritic spine enlargement and mouse behaviors.

## Materials and methods

### Animals and housing

All animal experiments were performed with the approval of and in strict accordance with the guidelines of the Academia Sinica Institutional Animal Care and Utilization Committee (protocol No 18-10-1234) and the Republic of China Council of Agriculture Guidebook for the Care and Use of Laboratory Animals. Pregnant rats and mice were housed individually and sacrificed using CO_2_ inhalation. Embryonic day E18.5 fetal pups of both sexes were sacrificed by decapitation for neuronal cultures. All animals were housed and bred in the animal facility of the Institute of Molecular Biology, Academia Sinica, under controlled humidity and temperature and a 12 h light/12 h dark cycle (light off at 19:00). Animals accessed water and food ad libitum. All efforts were made to minimize animal suffering and to reduce the number of animals utilized.

### Antibodies and reagents

AF polyclonal antibody was generated using synthetic peptide (amino acids 30-48) as antigen to immunize rabbit. Polyclonal HSP90 was a kind gift from Dr. Chung Wang [17]. Commercially available antibodies and chemicals used in this report are as follows: mouse monoclonal beta-actin (AC-74, mouse, 1:5,000); mouse monoclonal VCP (612183, 1:1000, BD Biosciences); mouse monoclonal Tubulin (TUB 2.1, T4026, 1:5000, Sigma); mouse monoclonal PSD-95 (K28/43, MABN68, 1:1000, Millipore); mouse monoclonal synaptophysin/SVP38 (S5768, 1:1000, Sigma); mouse monoclonal Myc (9B11, #2276, 1:1000, Cell Signaling Technology); rabbit polyclonal Myc (ab9106, 1:500, Abcam); chicken polyclonal GFP (ab13970, 1:5000, Abcam); mouse monoclonal GFP (JL-8, 632381, 1:2000, Clontech); vesicular glutamate transporter 1/vGLUT1 (AB5905, 1:1000, Millipore), Alexa Fluor-488- and -555-conjugated secondary antibodies (Invitrogen); phalloidin-Alexa Fluor-546 (A22283, 1:100, Thermo); phalloidin-Alexa Fluor-405 (A30104, 1:100, Thermo); and Latrunculin A (LatA, L5163, Sigma).

### Plasmid construction

For Myc-tagged AF expression plasmid, full-length rat AF (residue 1-640), N-terminal fragment AF-N (residue 1-300), and C-terminal fragment AF-C (residue 301-640) were PCR-amplified and subcloned into vector GW1-Myc2b digested by BglII and EcoRI. To generate AF-miR knockdown constructs, miRNA was designed using BLOCK-iT™ RNAi Designer (https://rnaidesigner.thermofisher.com/rnaiexpress/). According to the ranking score, the top two miRNAs (nucleotide residues 590-610 and nucleotide residues 709-729) were chosen, linked together, and cloned into pcDNA6.2-GW/EmGFP vector. Accordingly, this knockdown plasmid expresses two AF miRNAs, resulting in better knockdown efficiency. A control vector (pcDNA6.2–GW/EmGFP-miR, Invitrogen) expressing a miRNA predicted to not target any gene in mammalian genomes was used as the negative control in knockdown experiments. To generate the silent mutant resistant to AF-miR, site-directed mutagenesis was performed using the following oligonucleotide: 5′-GATTTGCTGATACGCACTCAT-3′ for nucleotide residues 590-610 and 5′-CTGGAACTTGTATCTAGTGAT-3′ for nucleotide residues 709-729, respectively. The underlined bases indicate the mutated sites. GFP-actin was purchased from Clontech and used to outline neuronal morphology.

### Hippocampal neuronal culture, transfection and immunofluorescence analysis

Hippocampal neurons isolated from E18-19 rat or mouse embryos were cultured and transfected as described previously [18–20]. For immunofluorescence analysis, neurons at a density of 300,000 cells per well were seeded onto coverslips (18 mm in diameter and 0.17–0.2 mm in thickness) coated with poly-L-lysine. Transfection using calcium phosphate precipitation was performed to deliver the plasmid DNA into neurons [18–20]. A total of 3 µg DNA was used for each well. Neurons were transfected at 6, 12 or 22 days in vitro (DIV) and immunostaining was performed at 12, 18 or 28 DIV to monitor dendritic arborization, dendritic spine formation and spine maintenance, respectively. Neurons were washed with phosphate-buffered saline (PBS) and fixed with 4% paraformaldehyde and 4% sucrose in PBS for 10 min, followed by permeabilization with cold methanol for 15 min (for SVP38) or 0.2% Triton X-100 in PBS at room temperature (for the other antibodies). After blocking with 10% bovine serum albumin (BSA), cells were incubated with primary antibodies diluted in PBS containing 3% BSA and 0.1% horse serum at 4 °C overnight, followed by extensive washes with PBS, and incubated with secondary antibodies conjugated with Alexa Fluor-488 and -555 for 2 h at room temperature. The images were acquired at room temperature using a confocal microscope (LSM700; Carl Zeiss) equipped with a 63 × /NA 1.4 oil objective lens, and Zen 2009 (Carl Zeiss) acquisition with Z series (1 µm/single stack) and analysis by ImageJ software (NIH). Super-resolution images were acquired using an LSM980 system with Airyscan2 (Plan-Apochromat 63 × /1.4 oil objective, 0.17 µm/single stack). For quantitation, the same set of experimental samples was acquired under the same microscope settings. Post-acquisition adjustment was avoided. To minimize personal bias, image acquisition was performed blind by having other lab members re-label the samples.

### Morphometry of dendritic arborization, dendritic protrusions and the distribution of synaptic proteins

For dendritic arborization analysis, cultured hippocampal neurons were transfected with GFP to outline neuronal morphology. Dendrite complexity was assessed by Sholl analysis (ImageJ) to count numbers of intersections [21–23]. For dendritic spine analysis, Z series confocal images were collected as described in the previous section. Each image consisted of 12–16 sections spaced 0.5 μm apart. The data were collected from two independent experiments for quantification. To characterize the morphometry of dendritic protrusions, 20 μm-long segments of dendrites starting 20 μm away from the soma were selected for analysis. Three parameters—including density, width and length of dendritic protrusions—were manually measured using ImageJ. To quantify the distribution and immunoreactivities of postsynaptic PSD-95 along dendritic protrusions, line scanning using ImageJ was performed. A 0.5 µm-wide line starting from the top of the dendritic protrusion and ending at the dendritic shaft was drawn to quantify the amount of proteins. Total immunoreactivities within 1 μm from the top of protrusions were also summed for statistical analysis. To quantify the presynaptic protein SVP38, a circle of 1 μm diameter was drawn at the tip of the dendritic protrusion, and immunoreactivities were quantified using ImageJ. Protrusions with a signal intensity of > 20 units were categorized as immunoreactivity-positive [24]. Functional synapses were defined by the colocalization of presynaptic vGLUT1 and postsynaptic PSD95. Overlap coefficients were analyzed using Zen 2009 (Carl Zeiss).

### Electrophysiology

Cultured hippocampal neurons at 18-19 DIV were used to record miniature EPSCs (mEPSCs) by whole-cell patch clamps. Neurons were incubated in extracellular solution, i.e. artificial cerebrospinal fluid (ACSF) solution containing 119 mM NaCl, 2.5 mM KCl, 1.3 mM MgSO_4_, 26.2 mM NaHCO_3_, 1 mM NaH_2_PO_4_, 2.5 mM CaCl_2_, 11 mM glucose, 0.001 mM tetrodotoxin, and 0.04 mM bicuculline. The intracellular solution contained 131 mM K-gluconate, 8 mM NaCl, 20 mM KCl, 10 mM HEPES, 2 mM Mg-ATP, 2 mM EGTA and 0.3 mM Na_3_GTP. Neurons were voltage-clamped at − 70 mV for recording with an Axon Axopatch 200B amplifier (Molecular Devices) and Digidata 1440A system (Molecular Devices), and then filtered at 1 kHz. Raw data were analyzed using Clampfit 10.7 software (Molecular Devices) and Mini Analysis (Syanptosoft). Statistical analyses of amplitude and frequency were performed using Prism.

### Generation of *klhl17* CRISPR-Cas9 knockout mice

*Klhl17* knockout (KO) mice were generated in C57BL/6 mice using a CRIPSR/Cas9 system. Two target sequences (gRNAs) were designed to eliminate the entire genome of *klhl17*: 5′-CTAACCTAGGCGCCACACGA (targeting the promoter region) and 5′-TAGACGTTGGAGTTCTATGG (targeting the region after exon 12). A T7 promoter sequence (5′-TTAATACGACTCACTATA) was added upstream of the gRNA sequences and a partial trans-activating crRNA sequence (5′-GTTTTAGAGCTAGAAATAGC) was added downstream of the gRNA sequence. After annealing and PCR-amplification, the product served as the in vitro transcription template to generate RNA for genomic editing. Mice carrying the mutant allele were backcrossed to wild-type C57BL/6 mice for at least five generations to remove potential off-target variants that might be introduced by CRISPR/Cas9. Genotyping was performed by genomic PCR using the primer set: 5′-TGTGGAAACAGGATGTGTGC-3′, 5′-CGCCTCATGCTCAAATATGC-3′ and 5′-AGGTCTGGGTTCTTGGTTCC-3′. The predicted PCR product sizes were 364 base pairs (bp) for the wild type (WT) allele and 500 bp for the KO allele. Immunoblotting using AF antibody was also performed to confirm *Klhl17* gene knockout.

### Transfection, immunoblotting and immunoprecipitation

Neuro-2A and COS1 cells were transfected with the indicated plasmids using Lipofectamine 2000 (Invitrogen) according to the manufacturer’s instructions. For immunoblotting analysis, 1 day after transfection, cells were washed with PBS twice and lysed directly with SDS sample buffer. Total cell lysates were separated using SDS-PAGE and analyzed using immunoblotting. For immunoprecipitation analysis, transfected Neuro-2A cells were washed with PBS and solubilized in lysis buffer (PBS pH 7.4, 1% Triton X-100, 2 mM PMSF, 2 μg/ml aprotinin, 2 μg/ml leupeptin, 2 μg/ml pepstatin and 10 μM MG132) at 4 °C for 30 min. The lysates were centrifuged at 15,000×*g* for 30 min to remove the cell debris. The solubilized extract was subjected to immunoprecipitation using Myc antibodies combined with protein A sepharose. After mixing by rotation at 4 °C overnight, the precipitates were sequentially washed twice with each of the following buffers: 1% Triton X-100/PBS; 0.5% NP-40/0.5 M LiCl/50 mM Tris (pH 8); 0.5 M LiCl/50 mM Tris (pH 8); 10 mM Tris (pH 8). The precipitated proteins were then analyzed by immunoblotting.

### Analysis of F-actin integrity and remodeling

To examine F-actin integrity, regular immunostaining was first performed, followed by staining with phalloidin-Alexa Fluor-546 or -405. Line scanning using ImageJ was performed to quantify the immunoreactivities of F-actin signal intensity in the dendritic protrusions. To explore F-actin remodeling, transfected COS1 cells were treated with 1 µM LatA for 30 min to disrupt F-actin filament and then replaced with regular culture medium for recovery for a further 60 min. F-actin remodeling was evaluated by measuring the area of whole cells, which were outlined by phalloidin-Alexa Fluor-546 and analyzed with ImageJ.

### Behavior

All of the behavioral tasks were performed using 2-month-old male mice habituated in the behavior room for at least one week prior to undertaking tasks. The behavior room was lit by fluorescent tubes with a light intensity of 240 lx.

*Open-field test* was performed based on previous studies [25]. For this assay, mice were individually placed into an open box (transparent plastic box 40 × 40 × 30 cm) to freely explore and they were recorded from above by videotaping for 10 min. The rearing number was counted manually. The moving distance was quantified using the Smart Video Tracking System (Panlab). Moving distance indicates horizontal locomotor activity. Rearing number indicates exploratory activity and vertical locomotor activity.

*Elevated plus maze test* was performed [25] using a maze consisting of two open arms (30 × 5 cm) and two enclosed arms (30 × 5 × 14 cm) that extended from a central platform (5 × 5 cm) elevated to a height of 45.5 cm above the floor. Mice were placed into the central area facing one open arm and allowed to explore the maze. Their movement was recorded by videotaping for 10 min. The time spent in the open arms, the closed arms, and the central area were quantified using the Smart Video Tracking System (Panlab).

*Reciprocal social interaction* (RSI) was performed as described previously [25, 26]. Test mice were individually isolated for a week before RSI. An unfamiliar mouse aged ~ 2 weeks younger than the test mouse was placed into the home cage of the test mouse for 10 min. The lid of the cage was removed and recording was carried out by videotaping from above under 400 lx illumination. Only the time the test mouse spent interacting with the unfamiliar mouse was manually recorded to represent RSI. Interactions initiated by the unfamiliar mouse were excluded from the analysis.

### Quantification and statistical analysis

The data were collected and analyzed blind by having the samples re-labeled by other lab members. For each experiment, 20 neurons were randomly collected from two independent experiments for quantification. To analyze dendritic spine density and morphology, three clearly recognizable dendrites of each neuron were subjected to quantification. To monitor protein distribution in or surrounding dendritic protrusions, 400 dendritic protrusions were analyzed for each group. Statistical analysis of spine density was performed with one-way ANOVA with Bonferroni t-test using GraphPad Prism 7.0. Width and length of dendritic protrusions shown in the cumulative probability distribution were analyzed with the Kolmogorov–Smirnov test (http://www.physics.csbsju.edu/stats/). A P-value of less than 0.05 was considered significant. All statistical methods and results are summarized in Additional file 1: Table S1.

## Results

### Subcellular expression of KLHL17/AF in neurons

Using immunoblotting with KLHL17/AF antibody, we first confirmed the previous observation [6, 7] that KLHL17/AF proteins are predominantly expressed in the brain (Fig. 1b). Immunostaining of mature neurons cultured in vitro for 18 days (18 DIV) revealed a pattern of KLHL17/AF puncta in the soma and along dendrites (Fig. 1c). High-magnification confocal imaging indicated that the KLHL17/AF puncta were distributed at dendritic spines as well as along the dendritic shaft, with these latter often being much larger than those at dendritic spines (Fig. 1d). To investigate the relative distribution of KLHL17/AF puncta and F-actin, we performed co-staining with phalloidin-Alexa Fluor-405. Super-resolution imaging using an LSM980 system with Airyscan2 revealed that KLHL17/AF immunoreactivity was colocalized either with or adjacent to F-actin at dendritic spines as well as in dendritic shafts (Fig. 1e). However, some KLHL17/AF puncta in dendritic shafts were not colocalized with or adjacent to F-actin (Fig. 1e). Thus, some KLHL17/AF proteins are associated with F-actin and some are independent of it.

### *Klhl17* knockdown impairs neuronal morphology

**Fig. 2:**
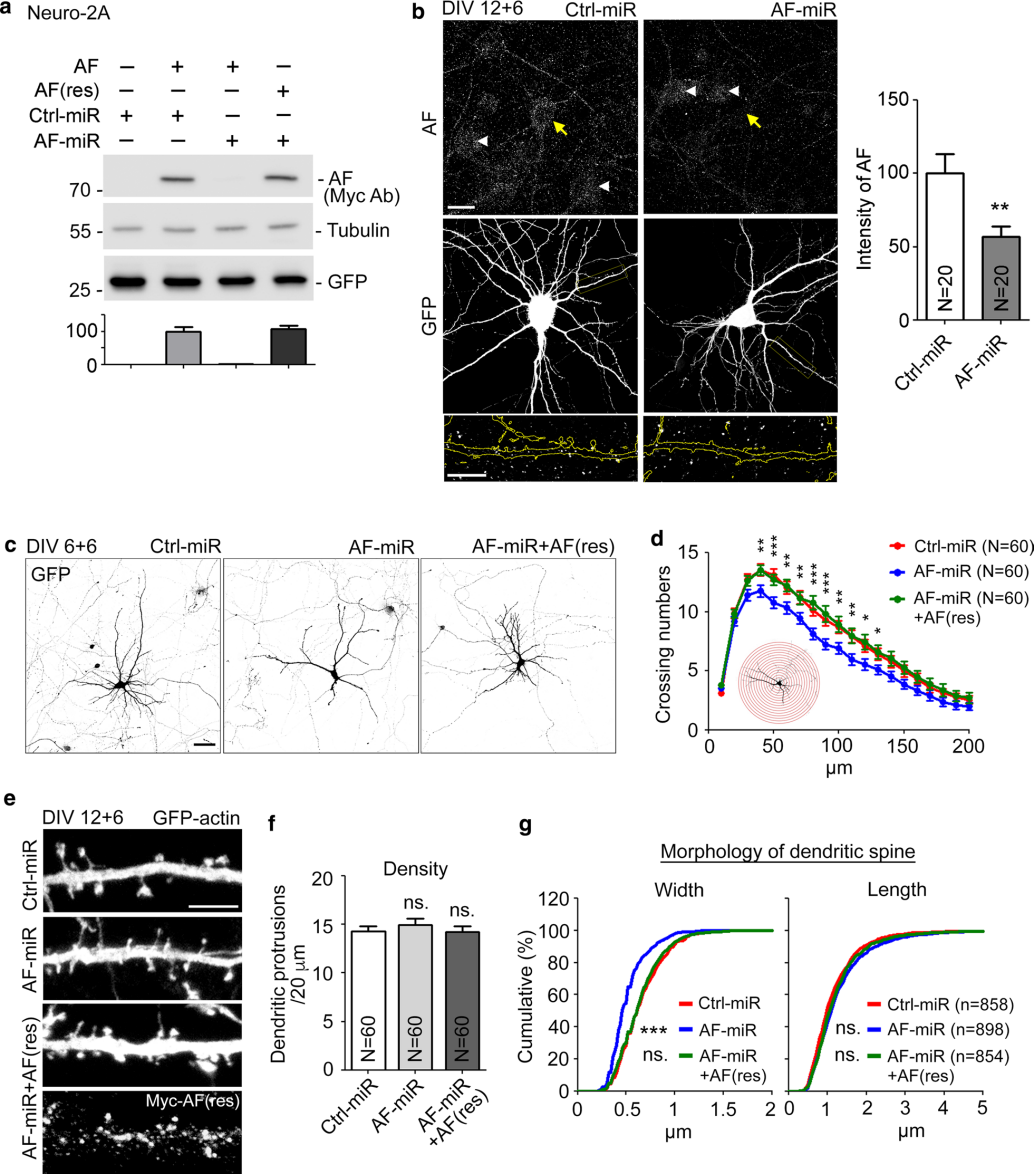
KLHL17/AF knockdown impairs dendritic arborization and spine formation. **a** The knockdown effect of AF-miR in Neuro-2A cells. Whole-cell extracts were collected and immunoblotted with Myc antibody to detect Myc-tagged AF and AF-miR-resistant AF-rescue construct (AF(res)). GFP and tubulin are loading controls. **b** AF-miR reduces endogenous KLHL17/AF protein levels in hippocampal neurons. Left: Cultured hippocampal neurons were transfected with the indicated plasmids at 12 DIV and harvested for immunostaining at 18 DIV. Since miRNA constructs coexpress GFP, GFP signals were used to outline miRNA-expressing neurons. Endogenous AF was detected using AF antibody. The AF signal in the soma was quantified. Yellow arrows and white arrowheads indicate transfected and non-transfected neurons, respectively. Enlarged images of dendrites (yellow box) are shown at bottom. Right: Quantification of knockdown efficiency. **c**–**g** Loss of KLHL17/AF impairs dendritic arborization (**c**–**d**) and spine formation (**e**–**g**). Cultured hippocampal neurons were transfected with the indicated plasmids at 6 (**c**–**d**) or 12 (**e**–**g**) DIV and then harvested for immunostaining at 12 (**c**–**d**) or 18 (**e**–**g**) DIV, respectively. AF knockdown reduces the complexity of dendrites and dendritic protrusion width, but not protrusion length or density. The AF-rescue construct AF(res) ameliorates the deficits of dendritic complexity and spine morphology caused by AF-miR. **c**, **e** Representative images. **e** The upper three panels show GFP signals. The lowest panel indicates expression of AF(res), as revealed by Myc tag antibody. **f** Quantification of protrusion density. **g** Quantification of the width and length of dendritic protrusions. N, the number of analyzed neurons (**a**, **d**) or dendrites (**f**); n, the number of analyzed dendritic protrusions. Samples were randomly collected from two independent experiments without knowing the treatment. Data represent the mean plus SEM. *P < 0.05; **P < 0.01; ***P < 0.001; ns, not significant. Scale bars: **b** 20 μm; enlarged inset, 10 μm; **c** 50 μm; **e** 5 μm. Unpaired t-test (**b)**, two-way ANOVA (**d**), one-way ANOVA (**f**), Kolmogorov–Smirnov test for cumulative probability (**g**)

Next, we investigated the role of KLHL17/AF in neuronal morphology. We expressed artificial miRNAs to knock down exogenous KLHL17/AF in mouse neuroblastoma Neuro-2A cells (Fig. 2a) and endogenous KLHL17/AF in cultured neurons (Fig. 2b). In Neuro-2A cells, expression of the *Klhl17* knockdown AF-miR construct effectively reduced KLHL17/AF protein levels (Fig. 2a). In cultured neurons, the immunoreactivity of endogenous KLHL17/AF was reduced by ~ 50% in the presence of AF-miR (Fig. 2b). Note that, similar to exogenous KLHL17/AF in cultured neurons, we found that endogenous KLHL17/AF also formed puncta in the soma and dendrites (Fig. 2b).

The AF-miR construct was then used to evaluate if KLHL17/AF regulates neuronal morphology. Transfection of AF-miR and vector control (Ctrl-miR) was performed at two time-points, i.e. 6 and 12 DIV, to assess the effect of AF-miR on dendritic arborization at 12 DIV and dendritic spine formation at 18 DIV, respectively. We found that KLHL17/AF is involved in dendritic arborization because knockdown of KLHL17/AF reduced intersection numbers, as established by Sholl analysis (Fig. 2c, d). When AF-miR was transfected at 12 DIV, only the width of dendritic spine heads was reduced at 18 DIV (Fig. 2e–g), suggesting that KLHL17/AF is required for enlargement of dendritic spine heads. These morphological effects were specifically caused by KLHL17/AF knockdown because coexpression of an AF(res) construct, a silent mutant resistant to AF-miR (Fig. 2a), rescued the dendritic arborization and width deficits induced by AF-miR at both 12 and 18 DIV (Fig. 2c–g). These results evidence a role for KLHL17/AF in controlling dendritic morphology, including dendritic arborization and dendritic spine formation.

**Fig. 3:**
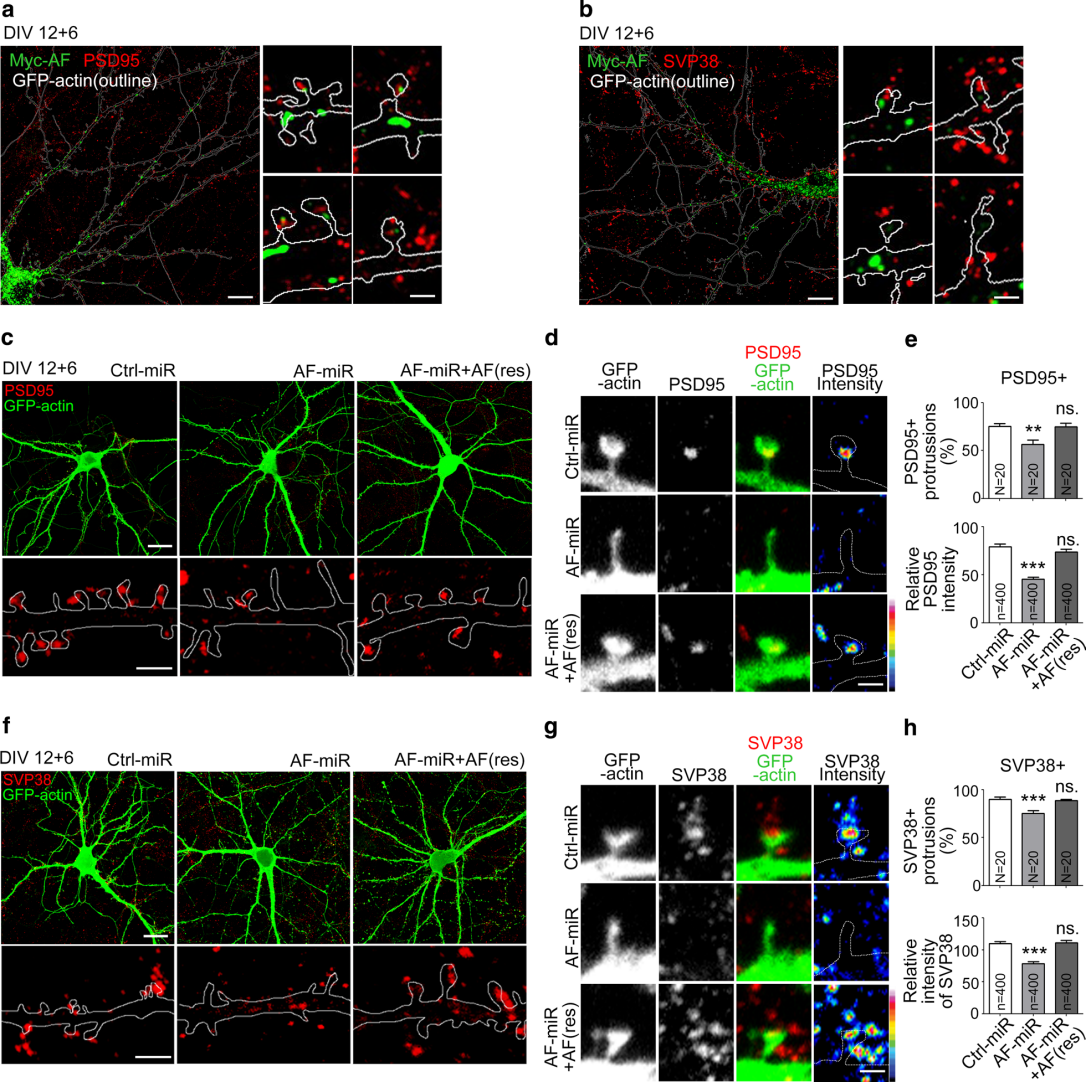
KLHL17/AF knockdown impairs functional synapse formation. Cultured hippocampal neurons were transfected with the indicated plasmids at 12 DIV and harvested for immunostaining using PSD95 (**a, c**–**e**) and SVP38 (**b, f**–**h**) antibodies at 18 DIV. Super-resolution images of AF distribution and that of synaptic proteins PSD95 (**a**) and SVP38 (**b**) in mature neurons, including whole cells and enlarged dendritic protrusions. **c**–**h** AF knockdown reduces functional synapse formation. **c**, **f** Representative confocal images, including whole cells and enlarged dendrite segments. **d**, **g** Representative confocal images of dendritic protrusions and heat maps. **e**, **h** Quantification of **e** PSD95- or **h** SVP38-positive protrusions and relative signal intensities. Samples were randomly collected from two independent experiments without knowing the treatment. N, the number of analyzed dendrites; n, the number of analyzed dendritic protrusions. Data represent the mean plus SEM. **P < 0.01; ***P < 0.001; ns, not significant. Scale bars: **a**, **b** whole cell: 10 μm, enlarged inset: 1 μm; **c**, **f** whole cell: 20 μm, enlarged inset: 2 μm; **d**, **g** 1 μm. One-way ANOVA (**e**, **h**).

Since the size of dendritic spine heads was reduced by KLHL17/AF knockdown, it likely influences the formation of functional synapses. To investigate that possibility, first we monitored the relative localizations of KLHL17/AF and the postsynaptic marker PSD95 and the presynaptic marker synaptophysin (SVP38) using super-resolution imaging. Although both KLHL17/AF and PSD95 are present in dendritic spines, there was no obvious colocalization of KLHL17/AF and PSD95 (Fig. 3a). Similarly, we did not detect colocalization between KLHL17/AF and SVP38 (Fig. 3b). We then monitored the expression levels of post- and pre-synaptic markers at synapses. In Ctrl-miR-expressing neurons, PSD95 puncta were clearly present at dendritic spines (Fig. 3a, b). However, in AF-miR-expressing neurons, we found that numbers of PSD95^+^ protrusions were lower than in Ctrl-miR-transfected neurons and that PSD95 signal intensity in PSD95^+^ protrusions was also reduced (Fig. 3c–e). Coexpression of AF(res) with AF-miR rescued PSD95 expression at dendritic spines (Fig. 3c–e), supporting that KLHL17/AF knockdown specifically reduced PSD95. For a presynaptic marker, we monitored expression of SVP38. Similar to PSD95, SVP38^+^ protrusions became fewer and the SVP38 signal intensity of individual protrusions was reduced upon KLHL17/AF knockdown (Fig. 3f–h). Together, these results indicate that KLHL17/AF knockdown reduces the protein machinery at synapses for neurotransmission via an indirect mechanism.

### KLHL17/AF is also required for dendritic spine maintenance

**Fig. 4:**
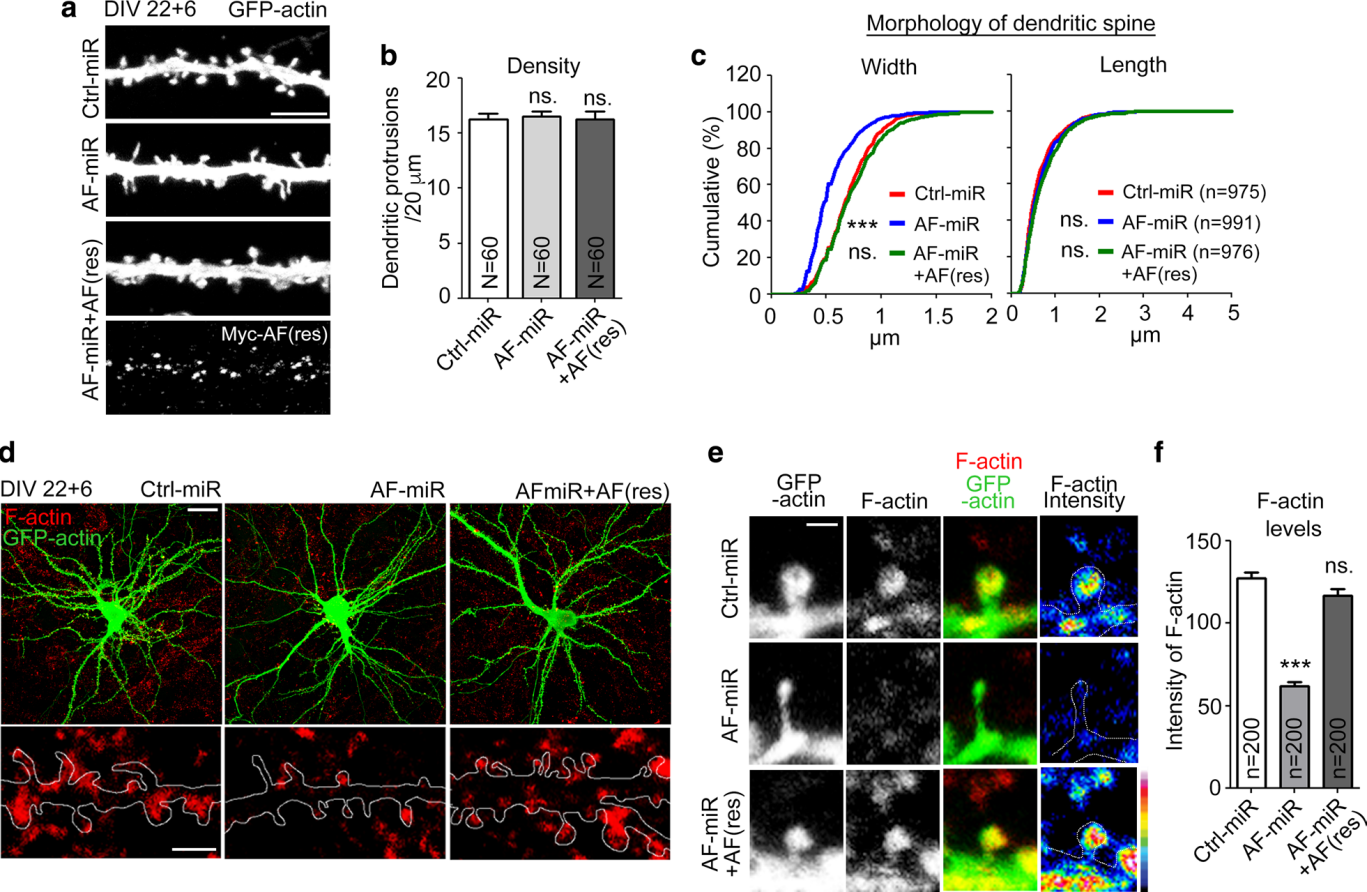
KLHL17/AF knockdown impairs dendritic spine maintenance and decreases F-actin intensity at dendritic spines. Cultured hippocampal neurons were transfected with the indicated plasmids at 22 DIV and harvested for immunostaining at 28 DIV. **a**–**c** AF knockdown impairs dendritic spine maintenance. AF knockdown reduces dendritic protrusion width, but not protrusion length or density. The AF-rescue construct AF(res) rescues the defect of dendritic spine morphology caused by AF-miR. **a** Representative images. **b** Quantification of protrusion density. **c** Quantification of the width and length of dendritic protrusions shown. **d**–**f** AF knockdown reduces the dendritic F-actin signal intensity in dendritic spines. Phalloidin staining was performed to label F-actin. **d** Representative images of whole cells and enlarged dendrite segments. **e** Representative images of dendritic protrusions. Heat maps show the intensities of F-actin. **f** Quantification of F-actin intensity at dendritic protrusions. Samples were randomly collected from two independent experiments without knowing the treatment. *N* the number of analyzed dendrites; n, the number of analyzed dendritic protrusions. Data represent the mean plus SEM. ***P < 0.001; *ns* not significant. Scale bars: **a** 5 μm; **d** whole cell, 20 μm; enlarged inset, 2 μm; **e** 1 μm. One-way ANOVA (**b**, **f**); Kolmogorov–Smirnov test for cumulative probability (**c**)

In addition to dendritic spine formation, we further investigated if KLHL17/AF is involved in dendritic spine maintenance. AF-miR and Ctrl-miR were delivered into cultured neurons at 22 DIV, i.e. when cultured neurons are fully mature. The effects of these treatments were then investigated at 28 DIV. Similar to the results at 18 DIV, only the width of dendritic spines, but not density or length of dendritic spines, was affected by AF-miR (Fig. 4a–c), suggesting that KLHL17/AF is also critical for maintaining the size of dendritic spines.

At 28 DIV, we further monitored F-actin distribution at dendritic spines. Phalloidin-Alexa Fluor-546 was included in immunostaining to label F-actin. We found that although GFP-actin still entered the dendritic spines of AF-miR-transfected neurons, signal of F-actin at dendritic spines was reduced by ~ 50% (Fig. 4d–f). Coexpression of AF(res) also rescued the distribution of F-actin in dendritic spines (Fig. 4d–f), again supporting the specificity of effect. Since F-actin forms the key cytoskeletal framework supporting dendritic spines, our study suggests that KLHL17/AF knockdown disrupts F-actin organization or remodeling, thereby inhibiting dendritic spine enlargement and synaptic protein distribution in cultured neurons.

### KLHL17/AF knockout also impairs enlargement of dendritic spines

**Fig. 5:**
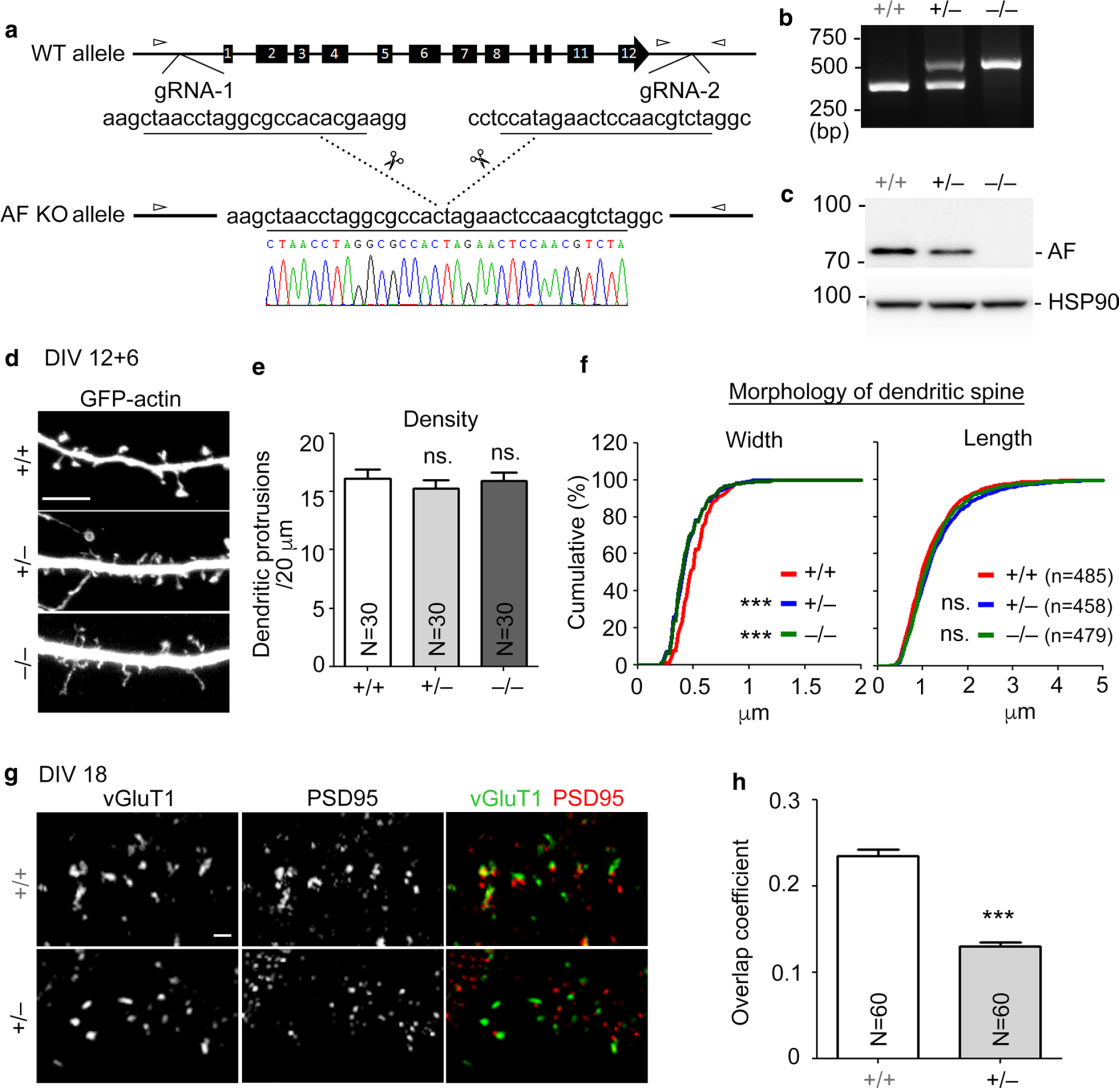
KLHL17/AF knockout mice show impairments of dendritic spinogenesis and functional synapse formation. **a** Generation of KLHL17/AF knockout mice by CRISPR/Cas9 technology. Schematic diagram of genomic editing using CRISPR/Cas9 gRNA to delete the *klhl17* gene. Small arrowheads indicate the position of primers for genomic PCR in (**b**). **b** Genomic PCR for genotyping. The sizes of PCR products were 364 bp for the WT allele and 500 bp for the KO allele. **c** Total cell extracts from cultured hippocampal neurons of different genotypes were collected at 18 DIV for immunoblotting. HSP90 was used as an internal control. **d**–**f** *Klhl17*^+*/–*^ and *Klhl17*^*–/–*^ mice exhibit impaired dendritic spinogenesis. Cultured hippocampal neurons of different genotypes were transfected with GFP-actin at 12 DIV and immunostained at 18 DIV. Cultured neurons from *Klhl17*^+*/–*^ and *Klhl17*^*–/–*^ mice exhibit narrow spine heads, but no change in spine length or spine density. **d** Representative images. **e** Quantification of protrusion density. **f** Quantification of width and length of dendritic protrusions. **g**, **h** *Klhl17*^+*/–*^ mice exhibit a reduced number of functional synapses. Cultured hippocampal neurons of *Klhl17*^+*/*+^ and *Klhl17*^+*/–*^ mice were immunostained for PSD95 and vGLUT1 at 18 DIV. **g** Representative images. **h** Quantification of overlap coefficients. Samples were randomly collected from two independent experiments without knowing the genotypes. *N* the number of analyzed dendrites; n, the number of analyzed dendritic protrusions. Data represent the mean plus SEM. ***P < 0.001; *ns* not significant. Scale bar: **d** 5 μm, **g** 1 μm. One-way ANOVA (**e**), unpaired t-test (**h**), Kolmogorov–Smirnov test for cumulative probability (**f**)

The entire *KLHL17* gene is deleted in IS patients [16]. To mimic the mutation possessed by patients, apart from RNA knockdown, we also employed CRISPR/Cas9 editing technology to knock out the *Klhl17* gene in mice (Fig. 5a). In addition to genomic PCR (Fig. 5b), immunoblotting using KLHL17/AF antibody also indicated that protein levels of KLHL17/AF were reduced in *Klhl17*^+*/–*^ neurons and completely absent in *Klhl17*^*–/–*^ neurons (Fig. 5c). When we compared *Klhl17*^+*/*+^, *Klhl17*^+*/–*^ and *Klhl17*^*–/–*^ hippocampal neurons, the width of dendritic spine heads of *Klhl17*^+*/–*^ and *Klhl17*^*–/–*^ neurons were noticeably reduced compared to *Klhl17*^+*/*+^ neurons (Fig. 5d–f), as observed upon KLHL17/AF knockdown. However, spine density and length were not altered by *Klkl17* deletion (Fig. 5d–f). We further used confocal imaging to analyze signal overlap of postsynaptic marker PSD95 and presynaptic marker vGLUT1 in *Klkl17*-deficient neurons. Consistent with the reduction of protein levels of pre- and post-synaptic markers in *Klhl17* knockdown neurons, the degree of PSD95 and vGLUT1 overlap was reduced in *Klhl17*^+*/–*^ neurons (Fig. 5g, h). Thus, the results of genetic deletion further support the specific role of KLHL17/AF in controlling enlargement of neuronal dendritic spine heads and formation of functional synapses.

### *Klhl17* deficiency alters synaptic activity and mouse behaviors

**Fig. 6:**
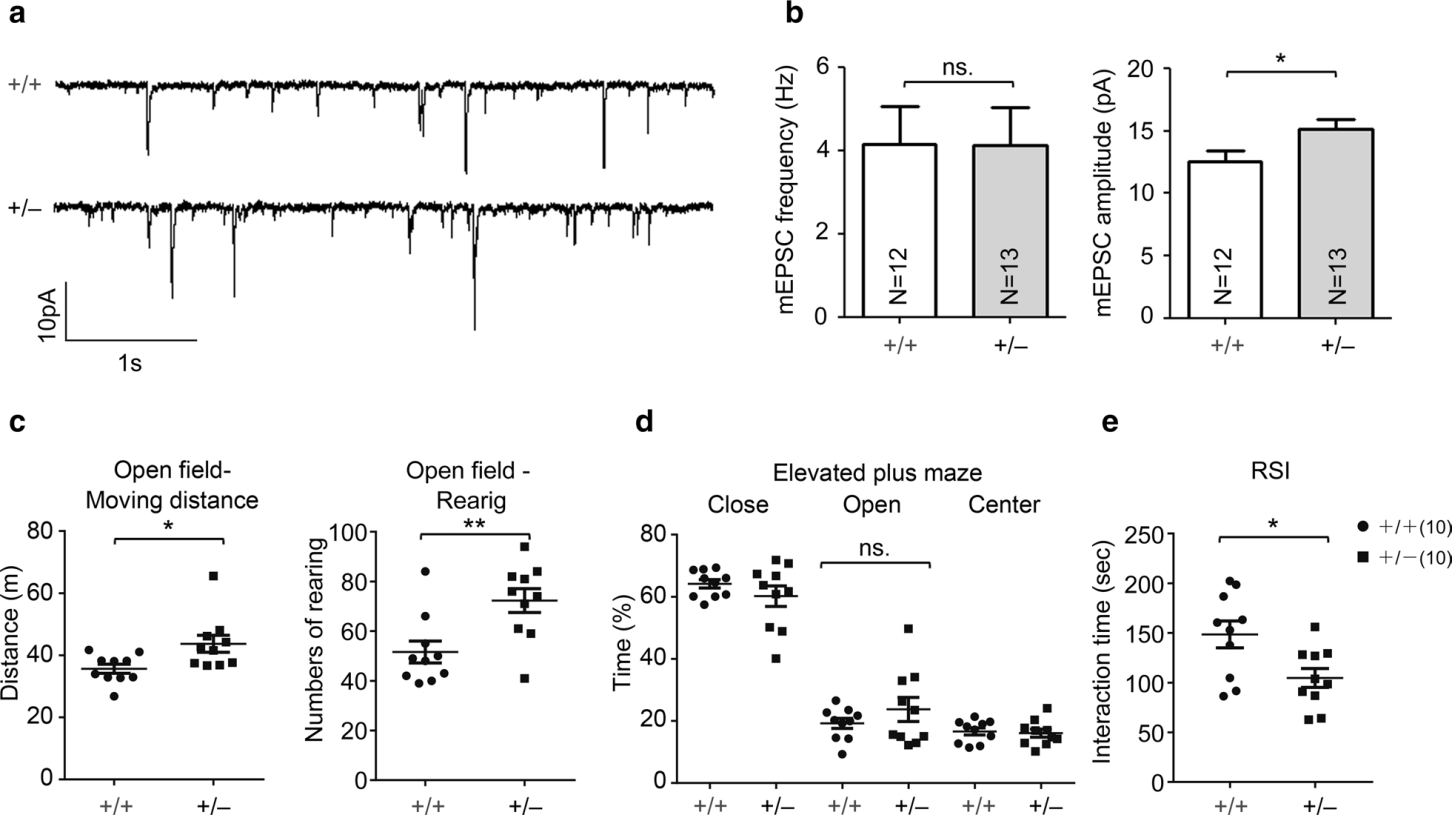
Electrophysiology analysis and behavior of *Klhl17*^+/–^ mice. **a**, **b** *Klhl17*^+/–^ mice exhibit increased mEPSC amplitudes. The mEPSCs of cultured hippocampal neurons were recorded at 18–19 DIV. *Klhl17*^+/–^ neurons show higher mEPSC amplitudes, but not mEPSC frequencies. **a** Representative mEPSC patterns. **b** Quantification of mEPSC frequency and amplitude. **c**–**e** *Klhl17*^+/–^ mice exhibit hyperactivity in an open field (**c**) and lower social interaction in the reciprocal social interaction test (**e**), but presented no difference to wild-type mice in the elevated plus maze (**d**). **a**, **b** Samples were randomly collected from four independent experiments without knowing the genotypes. *N* the number of analyzed neurons. Data represent the mean plus SEM. *P < 0.05; **P < 0.01; *ns* not significant. Scale bar: **a** 10 pA, 1 s. Unpaired t-test (**b**–**e**)

We further investigated if Klhl17 deficiency impairs neuronal activity and brain function. Electrophysiological recording was performed to measure mEPSC of cultured hippocampal neurons. Consistent with the results of dendritic spine analysis, we found that the mEPSC frequency of *Klhl17*^+*/–*^ neurons was comparable to that of WT neurons. However, mEPSC amplitude was slightly increased in *Klhl17*^+*/–*^ neurons (Fig. 6a, b), which might seem inconsistent with the reduction in dendritic spine head width (Fig. 5f). However, a previous study indicated that *Klhl17/Af* knockdown increased the expression levels of GLUR6, a subunit of the kainite receptor in cultured neurons [9], potentially explaining the slight increase in mEPSC amplitude of *Klhl17*^+*/–*^ neurons.

We then performed a series of behavioral assays—including open field, elevated plus maze and reciprocal social interaction—to evaluate the role of *Klhl17/Af* in brain function. Compared with WT mice, *Klhl17*^+*/–*^ mice exhibited longer moving distances and more frequent rearing behaviors in an open field (Fig. 6c), implying that *Klhl17* deficiency induced hyperactivity. There was no obvious difference between WT and *Klhl17*^+*/–*^ mice in our elevated plus maze assay (Fig. 6d). For reciprocal social interaction, *Klhl17*^+*/–*^ mice exhibited reduced interaction with unfamiliar mice (Fig. 6e). Taken together, the results of these behavioral assays support a role for KLHL17/AF in regulating brain function and mouse behaviors.

### Both N- and C-terminal halves of KLHL17/AF are required for F-actin remodeling

**Fig. 7:**
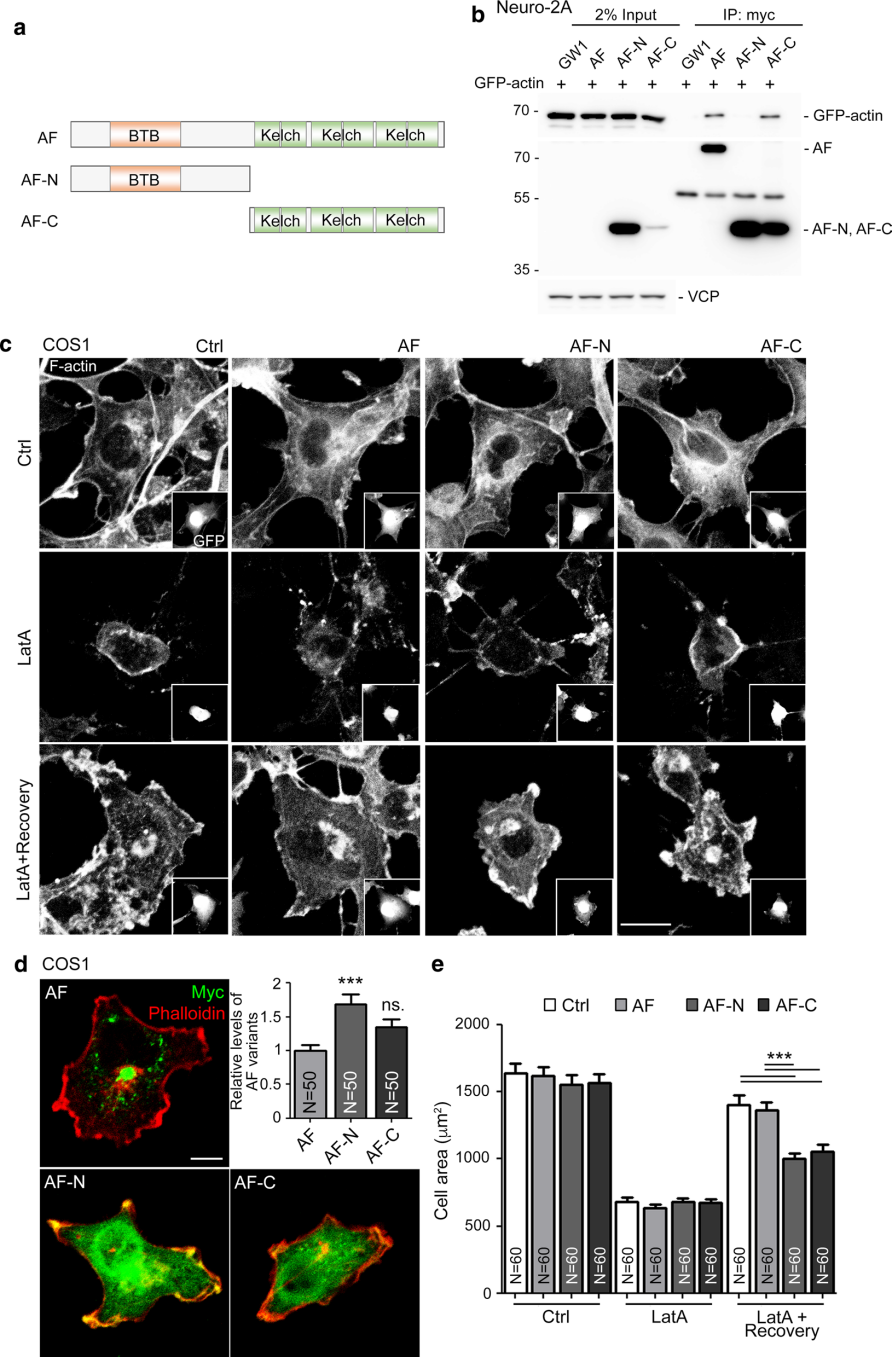
Both AF-N and AF-C are required for KLHL17/AF to regulate F-actin remodeling. **a** Schematic of AF-N and AF-C truncated mutants. AF-N consists of amino acid residues 1-300, which includes the BTB domain. AF-C comprises the C-terminal amino acid residues 301-640, which covers the six Kelch domains. All AF, AF-N and AF-C constructs were tagged with a Myc cassette at their N-terminal end for detection. **b** Actin-binding ability of AF variants. Neuro-2A cells were cotransfected with GFP-actin and different AF constructs, as indicated, and subjected to immunoprecipitation and immunoblotting using the indicated antibodies. Since protein levels of full-length AF and AF-C were very low in Neuro-2A lysates, they were barely detectable in 2% input lysate. However, all AF variants could be enriched after immunoprecipitation using Myc antibody. Note that even though AF-N was expressed at much higher levels in Neuro-2A cells, only full-length AF and AF-C were able to precipitate actin. **c**–**e** Both AF-N and AF-C alter F-actin reorganization ability. COS1 cells were cotransfected with GFP and AF variants, as indicated, and treated with Latrunculin A (LatA, 1 µM) for 30 min to disrupt F-actin filament. After treatment, cells were recovered for a further 60 min and subjected to immunostaining using Myc tag antibody to label AF expression and by phalloidin staining to label F-actin. **c** Representative images. GFP images are shown in insets to indicate transfected cells. **d** Expression patterns and levels of AF variants in COS1 cells. **e** Quantification of cell area before and after LatA treatment. Samples were randomly collected from two independent experiments without knowing the treatment. *N* the number of analyzed cells. Data represent the mean plus SEM. ***P < 0.001; *ns* non-significant. Scale bar: **c** 20 μm, **d** 10 μm. One-way ANOVA (**d**, **e**)

We then investigated how KLHL17/AF controls dendritic spine enlargement. Since the N- and C-terminal regions of KLHL17/AF possess distinct domains (Fig. 1a), we investigated which region is required for the functions of KLHL17/AF in dendritic spines. To do this, we generated an AF-N construct containing the BTB domain and an AF-C construct possessing all six Kelch domains (Fig. 7a). We first confirmed the interaction between KLHL17/AF and actin by means of co-immunoprecipitation. To do that, we co-transfected GFP-actin and Myc-tagged full-length AF, AF-N or AF-C into Neuro-2A cells. Cell lysates were then harvested for immunoprecipation using Myc-tag antibody. Although the expression levels of full-length AF and AF-C were much lower than that of AF-N (Fig. 7b), reasonable amounts of full-length AF and AF-C were still immunoprecipitated by Myc-tag antibody (Fig. 7b). Importantly, GFP-actin co-precipitated with full-length AF and AF-C, but not with AF-N (Fig. 7b), confirming the interaction between the Kelch domains of KLHL17/AF and actin.

Then, we investigated if AF-C alone alters F-actin organization. We included full-length AF and AF-N as controls. These constructs were co-transfected with GFP individually to outline cell morphology and label transfected cells, before monitoring F-actin organization by phalloidin staining. We did not observe obvious differences in cell morphology or F-actin organization among COS1 cells expressing full-length AF, AF-N or AF-C (Fig. 7c, upper). Latrunculin A (LatA; a reagent to depolymerize F-actin) treatment and washout was then performed to monitor if KLHL17/AF is involved in F-actin remodeling. We found that cell morphology and F-actin distribution were also comparable among the different groups upon LatA treatment (Fig. 7c, middle). However, after LatA washout and recovery for one hour, we observed that cells transfected with full-length AF and control vector had extended well to attach on the culture plate. However, AF-N- and AF-C-expressing cells were much smaller (Fig. 7c, lower). Similar to those in Neuro-2A cells, expression levels of AF-N were still higher than AF-C and full-length AF in COS1 cells (Fig. 7d). We also quantified cell area based on F-actin signal. Cell area was comparable among all groups in the absence of LatA treatment. However, LatA treatment reduced the size of all four groups of cells. Upon LatA washout, both AF-N- and AF-C-expressing cells were smaller than cells transfected with full-length AF and control vector (Fig. 7e).

### Both AF-N and AF-C regulate F-actin distribution at dendritic spines

**Fig. 8:**
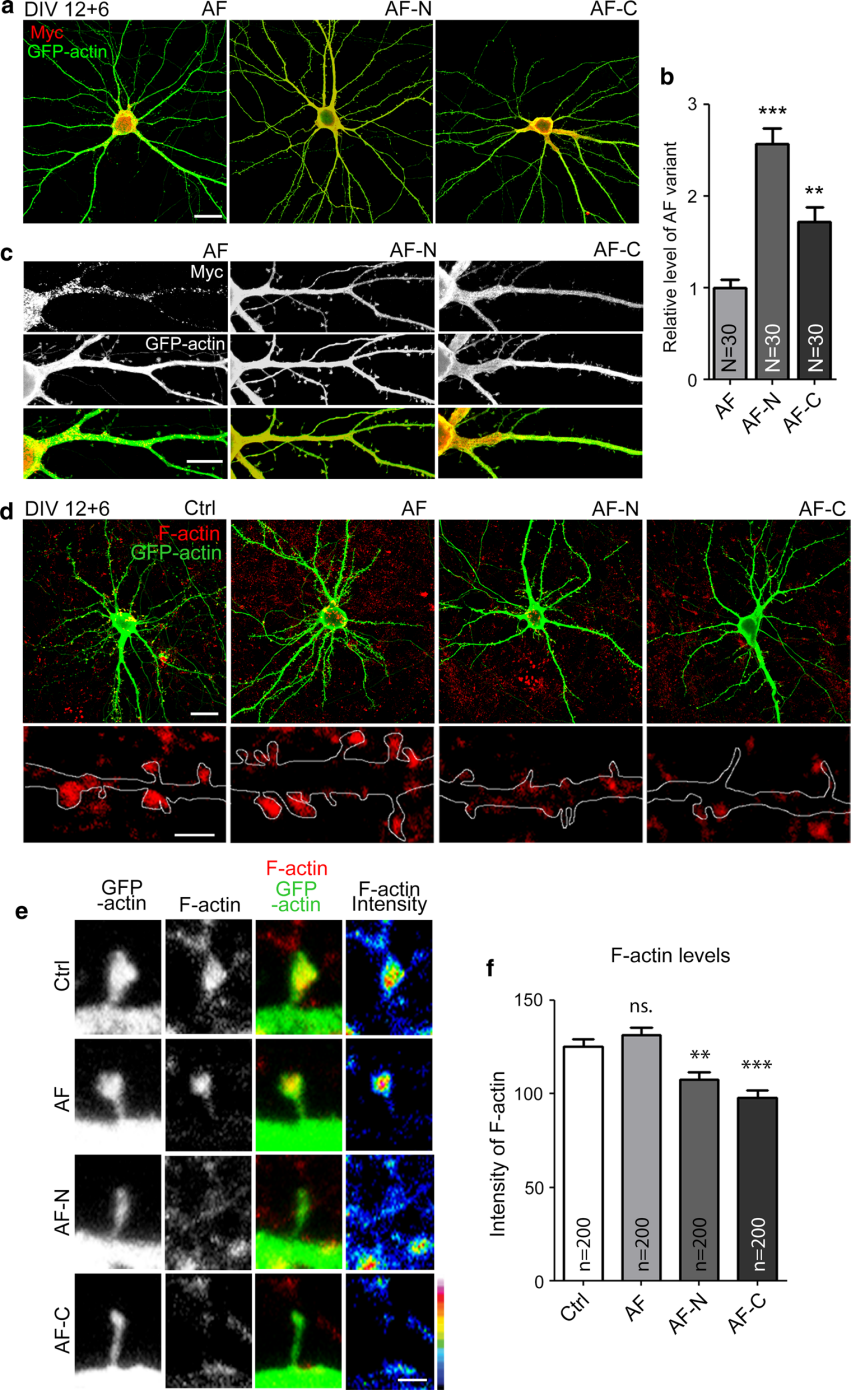
Truncated AF mutants reduce F-actin integrity at dendritic spines. Cultured hippocampal neurons were transfected with the indicated plasmids at 12 DIV and harvested for immunostaining at 18 DIV. **a**–**c** Expression patterns and levels of full-length AF, AF-N, AF-C, as revealed by Myc antibody. Full-length AF exhibits a punctate signal, but both AF-N and AF-C show diffuse patterns. **a** Representative images of whole cells. **b** Relative expression levels of AF variants in mature neurons. **c** Representative images of enlarged dendrite segments. **d**–**f** Truncated AF mutants reduce F-actin intensity in mature cultured neurons. Phalloidin staining was performed to assess F-actin integrity. **d** Representative images of whole cells and enlarged dendrite segments. **e** Representative images of dendritic protrusions. Heat maps show the intensities of F-actin. **f** Quantification of F-actin intensity at dendritic protrusions. *N* the number of analyzed neurons; *n* the number of analyzed dendritic protrusions. Samples were randomly collected from two independent experiments without knowing the treatment. Data represent the mean plus SEM. **P < 0.01; ***P < 0.001; ns, not significant. Scale bar: **a** 20 μm; **c** 10 μm; **d** whole cell: 20 μm, enlarged inset: 2 μm; **e** 1 μm. One-way ANOVA (**b**, **f**)

To determine if the AF-N and AF-C constructs could alter F-actin distribution in neuronal dendritic spines, Myc-tagged full-length AF, AF-N or AF-C were transfected into WT hippocampal neurons. We found that the dendritic arbors of AF-, AF-N- and AF-C-transfected mature neurons were not noticeably altered relative to vector control (Fig. 8a), though the hierarchy of expression levels (from high to low) of these three constructs remained AF-N > AF-C > AF (Fig. 8b). However, unlike for full-length AF-transfected neurons, we found that both AF-N- and AF-C-transfected neurons lacked the punctate pattern of KLHL17/AF, which was instead evenly distributed in neurons (Fig. 8c). Thus, both the N- and C-terminal regions of KLHL17/AF protein are required to generate the punctate pattern.

Phalloidin staining was then performed to monitor F-actin distribution at dendritic spines. Compared with vector control, F-actin signal intensity in dendritic spines harboring full-length AF did not increase further (Fig. 8d–f), suggesting that increased KLHL17/AF expression does not enhance synaptic targeting of F-actin. However, dendritic spines of AF-N- and AF-C-expressing neurons had much lower F-actin signals compared with vector control- and AF-transfected neurons (Fig. 8d–f). These results suggest that both the N- and C-terminal-truncated constructs of KLHL17/AF can act as dominant-negative mutants to inhibit F-actin distribution in neurons.

### Both N- and C-terminal regions of KLHL17/AF are essential for controlling dendritic spine enlargement

**Fig. 9:**
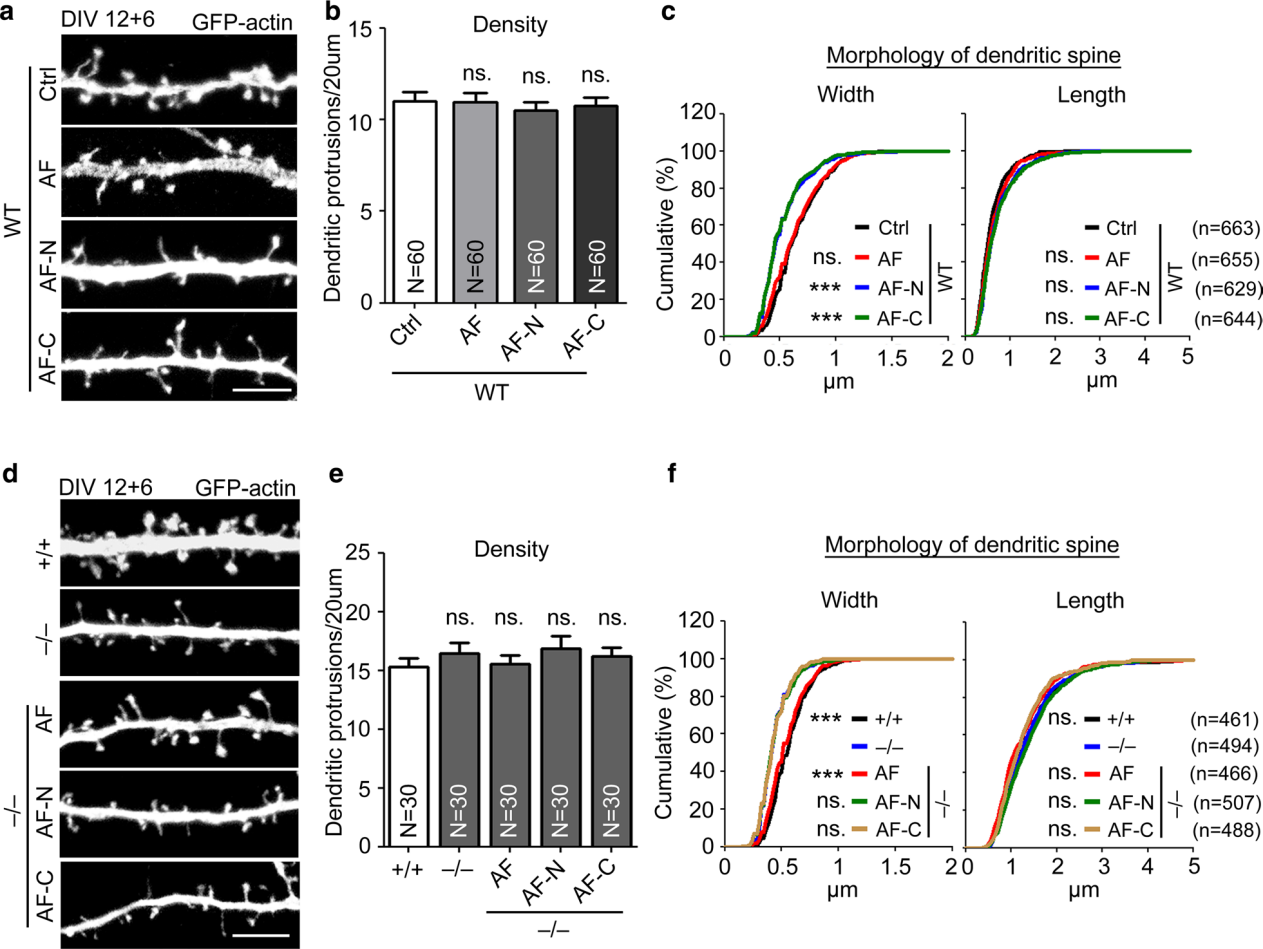
Both the N- and C-terminals of KLHL17/AF are required for dendritic spine enlargement. Cultured hippocampal neurons were transfected with the indicated plasmids at 12 DIV and harvested for immunostaining at 18 DIV. **a**–**c** Both AF-N and AF-C reduce the width of dendritic spines of WT cultured neurons. **d**–**f** Neither AF-N nor AF-C can rescue the impairment of dendritic spine enlargement in *Klhl17*^*–/–*^ cultured neurons. **a**, **d** Representative images. **b**, **e** Quantification of protrusion density. **c**, **f** Quantification of width and length of dendritic protrusions. *N* the number of analyzed dendrites; *n* the number of analyzed dendritic protrusions. Samples were randomly collected from two independent experiments without knowing the treatment. Data represent the mean plus SEM. ***P < 0.001; *ns* not significant. Scale bar: **a**, **d** 5 μm. One-way ANOVA (**b**, **e**). Kolmogorov–Smirnov test for cumulative probability (**c**, **f**)

Since dendritic spines are mainly supported by F-actin and given that the AF-N and AF-C constructs impaired F-actin distribution at dendritic spines, we assessed if the size of dendritic spines is affected by AF-N or AF-C expression. Indeed, expression of AF-N or AF-C in WT neurons reduced the width of dendritic spines (Fig. 9a–c), again indicating that these constructs act as dominant-negative mutants to impair dendritic spine enlargement. As observed in our knockdown and knockout experiments, the density and length of dendritic spines were not affected by expression of AF-N or AF-C (Fig. 9a–c), strengthening the evidence for a specific role of KLHL17/AF in dendritic spine enlargement.

To further confirm the function of KLHL17/AF in dendritic spine enlargement, we expressed full-length AF, AF-N and AF-C in *Klhl17*^*–/–*^ neurons. Full-length AF expression restored the size of dendritic spine heads in *Klhl17*^*–/–*^ neurons (Fig. 9d–f), confirming that the spine defect of *Klhl17*^*–/–*^ neurons is indeed caused by *Klhl17* deletion. Neither AF-N nor AF-C could restore dendritic spine head size of *Klhl17*^*–/–*^ neurons (Fig. 9d–f), indicating that the individual N- and C-terminal regions of KLHL17/AF alone cannot function appropriately to enlarge dendritic spine heads. Thus, both the N- and C-terminal regions of KLHL17/AF in the same molecule are necessary for it to exert its function.

## Discussion

**Fig. 10:**
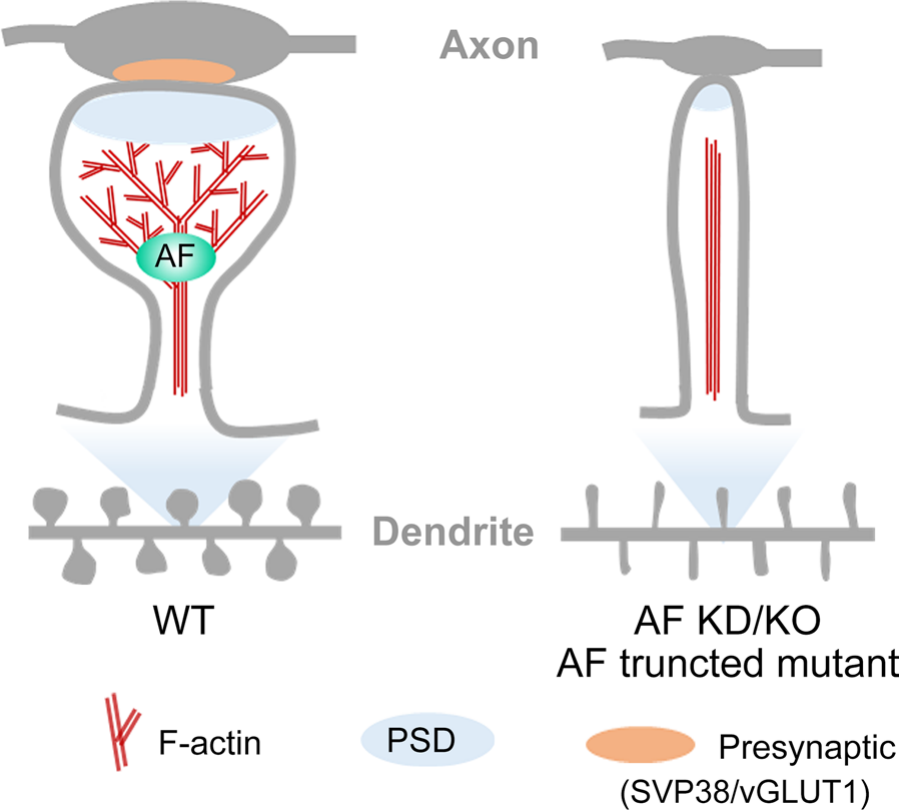
KLHL17/AF is critical for enlargement and maintenance of dendritic spines. Using a miRNA-based knockdown approach, *klhl17* knockout mice, and expression of truncated mutants, we show that KLHL17/AF deficiency impairs spinogenesis, functional synapse formation and F-actin remodeling

As a neuron-predominant actin-binding protein, we were intrigued to investigate the role of KLHL17/AF in neuronal morphogenesis, especially dendritic spine formation and plasticity. In this report, we show that KLHL17/AF controls the synaptic distribution of F-actin, as well as dendritic spine enlargement. We also show that it regulates the expression of post- and pre-synaptic markers, controls functional synapse formation and synaptic responses (Fig. 10). Behavioral assays further indicate that *Klhl17* deficiency results in hyperactivity and social interaction deficits. These results reveal a physiological role for KLHL17/AF and support the association of KLHL17/AF with neurological disorders.

Here, we show that KLHL17/AF, a protein predominantly expressed in neurons, specifically controls dendritic spine enlargement. Although all six actin-binding Kelch domains are present within the AF-C construct, we found that AF-C itself is not sufficient for F-actin enrichment at synapses or for dendritic spine enlargement. Instead, the N-terminal half of KLHL17/AF is also required. Since the N-terminal region of KLHL17/AF is involved in oligomerization [6], oligomerization by the N-terminal region potentially links multiple C-terminal Kelch repeats together for F-actin binding, bundling and branching, thereby securing the presence of F-actin at dendritic spines for spine head enlargement. Consistent with this speculation, we found that KLHL17/AF proteins tend to form small circular puncta, likely due to oligomerization. Moreover, these KLHL17/AF puncta are adjacent to or surrounded by F-actin at dendritic spines (Fig. 1e). Thus, it seems likely that KLHL17/AF nucleates multiple F-actin polymers at dendritic spines to support the morphology of spine heads. Future time-lapse recording to monitor the dynamics of KLHL17/AF and F-actin may provide evidence to address this possibility.

We previously investigated the function of another neuron-predominant cytoskeleton-binding protein, cortactin-binding protein-2 (CTTNBP2), in dendritic spine formation. Unlike KLHL17/AF, CTTNBP2 associates with both F-actin and microtubule, depending on the developmental stage [22, 27]. Before dendritic spines form, CTTNBP2 associates with microtubule along the dendritic shaft [22]. It then enters dendritic spines where it controls cortactin mobility to regulate dendritic spine formation and maintenance [28]. Interestingly, CTTNBP2 also forms dimer or oligomer via its N-terminal coiled-coil domain, and this oligomerization is required for the microtubule bundling mediated by the middle region of CTTNBP2 [22]. In contrast to KLHL17/AF, CTTNBP2 mainly regulates the density of dendritic spines, though some severe deficits identified from patients with autism spectrum disorders or due to complete knockout may also result in reduced spine width and length [27, 29]. Thus, the impact of CTTNBP2 deficiency on dendritic spines differs from that of KLHL17/AF. It would be intriguing to explore if there is any interrelationship between these two neuron-predominant cytoskeleton-associated proteins.

Apart from F-actin remodeling, KLHL17/AF also possesses another function in ubiquitin–proteasome protein degradation. To perform this function, KLHL17/AF uses its N-terminal region to interact with CUL3 E3 ubiquitin ligase and its C-terminal Kelch domains to interact with ubiquitination substrates [9]. Currently, it is unclear if its protein degradation activity is also required for how KLHL17/AF controls dendritic spine enlargement. Since KLHL17/AF has been shown to regulate the degradation of GLUR6 [9], it likely regulates synaptic activity via the CUL3-GLUR6 pathway. Consistent with this speculation, mEPSC amplitudes in *Klhl17*^+*/–*^ neurons were increased relative to WT neurons. It would also be interesting to investigate if the dual functions of KLHL17/AF are coordinated to control dendritic spine morphology and activity.

## Conclusions

Using knockdown and knockout approaches, as well as expression of truncated fragments, we have demonstrated physiological roles for KLHL17/AF in regulating dendritic spine enlargement, synaptic responses and mouse behaviors.

## Supplementary information

**Additional file 1: Table S1.** Table contains all statistical methods and results

## Data Availability

Data generated or analyzed during this study are included in this published article and its supplementary information files.

## Abbreviations

AF: Actinfilin
BTB: Broad-Complex, Tramtrack and Bric-a-brac
CTTNBP2: Cortactin-binding protein 2
Cul3: Cullin3
IS: Infantile spasms
KLHL17: Kelch-like 17
LatA: Latrunculin A
