# Supplementary material for: KLHL17/Actinfilin, a brain-specific gene associated with infantile spasms and autism, regulates dendritic spine enlargement
Source: J Biomed Sci. 2020 Dec 1;27:103. doi: 10.1186/s12929-020-00696-1 (PMC7708131; doi:10.1186/s12929-020-00696-1)
Supplement: Supplementary file 1 — Additional file 1: Table S1. Table contains all statistical methods and results [file 12929_2020_696_MOESM1_ESM.pdf]

Figure		Assay Performed	Parameter (Unit)	Groups	N	Descriptive Statistics Average ± SEM	Statistical Analysis					Column statistics		
							Statistical Test			Significance		Factor	Comparison	P value
	b	Knockdown efficiency of endogenous AF	Intensity of AF				Statistical Test		P value					
				Ctrl-miR	20	18.15±2.404	Unpaired T test		0.0067	t(38)=2.868	Two-tail			
				AF-miR	20	10.33±1.285								
									Two way ANOVA			Multiple Comparison Procedures (Bonferroni t-test)		
			No. of crossing intersection_ 40µm	Ctrl-miR	60	13.55±0.5149							Ctrl-miR vs. AFmiR	0.0033
				AF-miR	60	11.77±0.4918							Ctrl-miR vs. AF(res)	>0.9999
				AF(res)	60	13.53±0.4169								
			No. of crossing intersection_ 50µm	Ctrl-miR	60	13.12±0.5031							Ctrl-miR vs. AFmiR	<0.0001
				AF-miR	60	10.78±0.4964							Ctrl-miR vs. AF(res)	>0.9999
				AF(res)	60	12.71±0.4732								
			No. of crossing intersection_ 60µm	Ctrl-miR	60	12.06±0.5236							Ctrl-miR vs. AFmiR	0.0049
				AF-miR	60	10.34±0.4945							Ctrl-miR vs. AF(res)	>0.9999
				AF(res)	60	12.23±0.5277								
			No. of crossing intersection_ 70µm	Ctrl-miR	60	11.23±0.5358							Ctrl-miR vs. AFmiR	0.0033
				AF-miR	60	9.45±0.4956							Ctrl-miR vs. AF(res)	>0.9999
				AF(res)	60	11.14±0.5386								
		Cultured neuron transfected with Ctrl-miR, AF-miR, AF(res)_DIV 6+6_Sholl analysis	No. of crossing intersection_ 80µm	Ctrl-miR	60	10.14±0.4928							Ctrl-miR vs. AFmiR	0.0006
				AF-miR	60	8.117±0.4865							Ctrl-miR vs. AF(res)	0.7006
	d			AF(res)	60	10.79±0.5907	Genotype:: F(2,6661)=56.55, P<0.0001 intersection::F(39,6661)=286, P<0.0001 F(78,6661)=1.561, P=0.0012				Crossing Interaction::			
			No. of crossing intersection_ 90µm	Ctrl-miR	60	9.317±0.5239							Ctrl-miR vs. AFmiR	0.0004
				AF-miR	60	7.242±0.4599							Ctrl-miR vs. AF(res)	>0.9999
				AF(res)	60	9.825±0.6654								
2			No. of crossing intersection_ 100µm	Ctrl-miR	60	8.708±0.5502							Ctrl-miR vs. AFmiR	0.0029
				AF-miR	60	6.908±0.4904							Ctrl-miR vs. AF(res)	>0.9999
				AF(res)	60	8.942±0.6675								
			No. of crossing intersection_ 110µm	Ctrl-miR	60	7.917±0.5346							Ctrl-miR vs. AFmiR	0.0011
				AF-miR	60	5.975±0.4642							Ctrl-miR vs. AF(res)	>0.9999
				AF(res)	60	7.975±0.6436								
			No. of crossing intersection_ 120µm	Ctrl-miR	60	7.133±0.5226							Ctrl-miR vs. AFmiR	0.0117
				AF-miR	60	5.558±0.4757							Ctrl-miR vs. AF(res)	>0.9999
				AF(res)	60	7.45±0.6263								
			No. of crossing intersection_ 130µm	Ctrl-miR	60	6.408±0.5108							Ctrl-miR vs. AFmiR	0.0477
				AF-miR	60	5.092±0.4182							Ctrl-miR vs. AF(res)	>0.9999
				AF(res)	60	6.592±0.6017								
									One way ANOVA			Multiple Comparison Procedures (Bonferroni t-test)		
	f		No. of protrusions / 20um	Ctrl-miR	60	14.32±0.5073							Ctrl-miR vs. AFmiR	>0.999
				AF-miR	60	14.97±0.612			F(2,177)=0.5132, P=0.5994				Ctrl-miR vs. AF(res)	>0.999
				AF(res)	60	14.23±0.557								
	g1	Cultured neuron transfected with Ctrl-miR, AF-miR, AF(res)_DIV 12+6	Cumulative (%) in spine width				Statistical Test		P value	D				
				Ctrl-miR	858		KS-test	Ctrl-miR vs. AFmiR	<0.0001	0.3137				
				AF-miR	898		KS-test	Ctrl-miR vs. AF(res)	0.3	0.0468				
				AF(res)	854									
	g2		Cumulative (%) in spine length				Statistical Test		P value	D				
				Ctrl-miR	858		KS-test	Ctrl-miR vs. AFmiR	0.124	0.0811				
				AF-miR	898		KS-test	Ctrl-miR vs. AF(res)	0.525	0.0568				
				AF(res)	854									
									One way ANOVA			Multiple Comparison Procedures (Bonferroni t-test)		
	e1		Percentage (%) of PSD95+ protrusion/Dendrite	Ctrl-miR	20	75.25±2.844							Ctrl-miR vs. AFmiR	0.0021
				AF-miR	20	56.5±4.429			F(2,57)=8.435, P=0.0006				Ctrl-miR vs. AF(res)	>0.999
				AF(res)	20	75±3.663								
									One way ANOVA			Multiple Comparison Procedures (Bonferroni t-test)		
	e2		Intensity of PSD95/protrusion	Ctrl-miR	400	79.17±2.91							Ctrl-miR vs. AFmiR	<0.0001
		Cultured neuron transfected with Ctrl-miR, AF-miR, AF(res)_DIV 12+6		AF-miR	400	45.77±1.949			F(2,1197)=49.55, P<0.0001				Ctrl-miR vs. AF(res)	0.3912
				AF(res)	400	73.72±2.68								
3									One way ANOVA			Multiple Comparison Procedures (Bonferroni t-test)		
	h1		Percentage (%) of SVP38+ protrusion/Dendrite	Ctrl-miR	20	90±2.46							Ctrl-miR vs. AFmiR	<0.0001
				AF-miR	20	75.25±2.844			F(2,57)=12.89, P<0.0001				Ctrl-miR vs. AF(res)	>0.999
				AF(res)	20	88.75±1.196								
									One way ANOVA			Multiple Comparison Procedures (Bonferroni t-test)		
	h2		Intensity of SVP38/protrusion	Ctrl-miR	400	109.6±3.204							Ctrl-miR vs. AFmiR	<0.0001
				AF-miR	400	78.68±3.098			F(2,1197)=33.3, P<0.0001				Ctrl-miR vs. AF(res)	>0.999

				AF(res)	400	111.4±3.261							
4	b	Cultured neuron transfected with Ctrl-miR, AF-miR, AF(res)_DIV 22+6	No. of protrusions / 20um						One way ANOVA			Multiple Comparison Procedures (Bonferroni t-test)	
				Ctrl-miR	60	16.25±0.5249						Ctrl-miR vs. AFmiR	>0.999
				AF-miR	60	16.52±0.492			F(2,177)=0.06293, P=0.939			Ctrl-miR vs. AF(res)	>0.999
				AF(res)	60	16.28±0.6991							
	c1		Cumulative (%) in spine width				Statistical Test		P value	D			
				Ctrl-miR	975		KS-test	Ctrl-miR vs. AFmiR	<0.0001	0.3498			
				AF-miR	991		KS-test	Ctrl-miR vs. AF(res)	0.056	0.0639			
				AF(res)	976								
	c2		Cumulative (%) in spine length				Statistical Test		P value	D			
				Ctrl-miR	975		KS-test	Ctrl-miR vs. AFmiR	0.062	0.0591			
				AF-miR	991		KS-test	Ctrl-miR vs. AF(res)	0.056	0.06			
				AF(res)	976								
	f		Intensity of F-actin/protrusion						One way ANOVA			Multiple Comparison Procedures (Bonferroni t-test)	
				Ctrl-miR	200	127.2±3.589						Ctrl-miR vs. AFmiR	<0.0001
				AF-miR	200	61.6±2.922			F(2,597)=105.5, P<0.0001			Ctrl-miR vs. AF(res)	0.101
				AF(res)	200	116.9±3.737							
5	e	Cultured neuron with different genotype_DIV 12+6	No. of protrusions / 20um						One way ANOVA			Multiple Comparison Procedures (Bonferroni t-test)	
				klhl17 +/+	30	16.17±0.7537						klhl17 +/+ vs. klhl17 +/-	>0.999
				klhl17 +/-	30	15.27±0.767			F(2,87)=0.4062, P=0.6674			klhl17 +/+ vs. klhl17 -/-	>0.999
				klhl17 -/-	30	15.97±0.7022							
	f1		Cumulative (%) in spine width				Statistical Test		P value	D			
				klhl17 +/+	485		KS-test	klhl17 +/+ vs. klhl17 +/-	<0.0001	0.2475			
				klhl17 +/-	458		KS-test	klhl17 +/+ vs. klhl17 -/-	<0.0001	0.2794			
				klhl17 -/-	479								
	f2		Cumulative (%) in spine length				Statistical Test		P value	D			
				klhl17 +/+	485		KS-test	klhl17 +/+ vs. klhl17 +/-	0.098	0.0794			
				klhl17 +/-	458		KS-test	klhl17 +/+ vs. klhl17 -/-	0.333	0.0604			
				klhl17 -/-	479								
	h	Cultured neuron with different genotype_DIV 18	Overlap coefficient				Statistical Test		P value				
				klhl17 +/+	60	).2354±0.00700	Unpaired T test		p<0.0001	t(118)=12.27	Two-tail		
				klhl17 +/-	60	).1301±0.00495							
6	b1	Cultured neuron with different genotype_DIV 18-19	mEPSC frequency				Statistical Test		P value				
				klhl17 +/+	12	4.157±0.9095	Unpaired T test		0.9852	t(23)=0.01874	Two-tail		
				klhl17 +/-	13	4.133±0.9053							
	b2		mEPSC amplitude				Statistical Test		P value				
				klhl17 +/+	12	12.59±0.8143	Unpaired T test		0.0315	t(23)=2.29	Two-tail		
				klhl17 +/-	13	15.15±0.7697							
	c1	Open field	Moving distance				Statistical Test		P value				
				klhl17 +/+	10	3566±145.3	Unpaired T test		0.0186	t(18)=2.587	Two-tail		
				klhl17 +/-	10	4369±274.5							
	c2		Rearing				Statistical Test		P value				
				klhl17 +/+	10	51.6±4.41	Unpaired T test		0.0053	t(18)=3.173	Two-tail		
				klhl17 +/-	10	72.3±4.808							
	d	Elevated plus maze	Open arm				Statistical Test		P value				
				klhl17 +/+	10	19.22±1.624	Unpaired T test		0.2953	t(18)=1.078	Two-tail		
				klhl17 +/-	10	23.73±3.864							
	e	Reciprocal social interaction	Interaction time				Statistical Test		P value				
				klhl17 +/+	10	148.5±13.48	Unpaired T test		0.0164	t(18)=2.645	Two-tail		
				klhl17 +/-	10	104.8±9.574							
7	d	AF, AF-N, AF-C expressed COS1	Expression level of AF variants						One way ANOVA			Multiple Comparison Procedures (Bonferroni t-test)	
				AF	50	1±0.08289						AF vs. AF-N	0.0003
				AF-N	50	1.684±0.1447			F(2,147)=8.122, P=0.0005			AF vs. AF-C	0.1368
				AF-C	50	1.342±0.1242							
	e	AF, AF-N, AF-C expressed COS1 with LatA treatment_F-actin reorganization	Cell area (LatA+ Recovery)						One way ANOVA			Multiple Comparison Procedures (Bonferroni t-test)	
				Ctrl	60	1404±72.79						Ctrl vs. AF-N	<0.0001
				AF	60	1363±59.18						Ctrl vs. AF-C	0.0001
				AF-N	60	1003±40.36			F(3,236)=13.27, P<0.001			AF vs. AF-N	<0.0001
				AF-C	60	1056±49.15						AF vs. AF-C	0.0001
	b	Cultured neuron	Expression level of AF variants						One way ANOVA			Multiple Comparison Procedures (Bonferroni t-test)	
				AF	30	1±0.09282						AF vs. AF-N	<0.0001
				AF-N	30	2.567±0.9621			F(2,87)=28.82, P<0.0001			AF vs. AF-C	0.0024
				AF-C	30	1.719±0.1568							

8		transfected with AF, AF-N, AF-C_DIV 12+6					One way ANOVA				Multiple Comparison Procedures (Bonferroni t-test)	
	f		Intensity of F-actin/protrusion	Ctrl	200	125.5±3.608					Ctrl vs. AF	>0.999
				AF	200	131.7±3.791	F(3,796)=16.52, P<0.0001				Ctrl vs. AF-N	0.0056
				AF-N	200	107.5±3.943					Ctrl vs. AF-C	<0.0001
				AF-C	200	98.06±3.992						
							One way ANOVA				Multiple Comparison Procedures (Bonferroni t-test)	
	b		No. of protrusions / 20um	Ctrl	60	11.05±0.463					Ctrl vs. AF	>0.999
				AF	60	10.95±0.5242	F(3,236)=0.2354, P=0.8716				Ctrl vs. AF-N	>0.999
				AF-N	60	10.52±0.4618					Ctrl vs. AF-C	>0.999
				AF-C	60	10.75±0.4915						
							Statistical Test		P value	D		
	c1	Cultured neuron transfected with AF, AF-N, AF-C_DIV 12+6	Cumulative (%) in spine width	Ctrl	663		KS-test	Ctrl vs. AF	0.055	0.0733		
				AF	655		KS-test	Ctrl vs. AF-N	<0.0001	0.3004		
				AF-N	629		KS-test	Ctrl vs. AF-C	<0.0001	0.3147		
				AF-C	644							
							Statistical Test		P value	D		
	c2		Cumulative (%) in spine length	Ctrl	663		KS-test	Ctrl vs. AF	0.115	0.0654		
				AF	655		KS-test	Ctrl vs. AF-N	0.064	0.0717		
				AF-N	629		KS-test	Ctrl vs. AF-C	0.061	0.0714		
				AF-C	644							
							One way ANOVA				Multiple Comparison Procedures (Bonferroni t-test)	
9	e		No. of protrusions / 20um	klhl17 +/+ ; Ctrl	30	15.37±0.6806					klhl17 +/+ ; Ctrl vs.	>0.999
				klhl17 -/- ; Ctrl	30	16.47±0.885					klhl17 +/+ ; Ctrl vs.	>0.999
				klhl17 -/- ; AF	30	15.53±0.786	F(4,145)=0.5755, P=0.6808				klhl17 +/+ ; Ctrl vs.	>0.999
				klhl17 -/- ; AF-N	30	16.9±1.069					klhl17 +/+ ; Ctrl vs.	>0.999
				klhl17 -/- ; AF-C	30	16.23±0.7606						
							Statistical Test		P value	D		
	f1	klhl17 -/- cultured neuron transfected with AF, AF-N, AF-C_DIV 12+6	Cumulative (%) in spine width	klhl17 +/+ ; Ctrl	461		KS-test	klhl17 +/+ ; Ctrl vs.	<0.0001	0.3643		
				klhl17 -/- ; Ctrl	494		KS-test	klhl17 -/- ; Ctrl vs.	<0.0001	0.2845		
				klhl17 -/- ; AF	466		KS-test	klhl17 -/- ; Ctrl vs.	0.985	0.0286		
				klhl17 -/- ; AF-N	507		KS-test	klhl17 -/- ; Ctrl vs.	0.967	0.0314		
				klhl17 -/- ; AF-C	488							
							Statistical Test		P value	D		
	f2		Cumulative (%) in spine length	klhl17 +/+ ; Ctrl	461		KS-test	klhl17 +/+ ; Ctrl vs.	0.717	0.0447		
				klhl17 -/- ; Ctrl	494		KS-test	klhl17 -/- ; Ctrl vs.	0.082	0.0798		
				klhl17 -/- ; AF	466		KS-test	klhl17 -/- ; Ctrl vs.	0.348	0.0585		
				klhl17 -/- ; AF-N	507		KS-test	klhl17 -/- ; Ctrl vs.	0.628	0.047		
				klhl17 -/- ; AF-C	488							
